# Supplementary material for: Molecular basis of ligand binding and receptor activation at the human A3 adenosine receptor
Source: Nat Commun. 2025 Aug 18;16:7674. doi: 10.1038/s41467-025-62872-x (PMC12361428; doi:10.1038/s41467-025-62872-x)
Supplement: Supplementary file 1 — Supplementary Information [file 41467_2025_62872_MOESM1_ESM.pdf]

Supplementary Materials for

Molecular basis of ligand binding and receptor activation at the human A_3 adenosine receptor

Liudi Zhang[1,2,†], Jesse I. Mobbs[1,2,†], Felix M. Bennetts[1,2], Hariprasad Venugopal[3], Anh T.N. Nguyen[1], Arthur Christopoulos[1,2,4], Daan van der Es[5], Laura H. Heitman[5,6], Lauren T. May[1,*], Alisa Glukhova[1,2,7,8,*], and David M. Thal[1,2,*]

[1]Drug Discovery Biology, Monash Institute of Pharmaceutical Sciences, Monash University; Parkville, Australia.
[2]ARC Centre for Cryo-electron Microscopy of Membrane Proteins, Monash Institute of Pharmaceutical Sciences, Monash University; Parkville, Australia.
[3]Ramaciotti Centre for Cryo-Electron Microscopy, Monash University; Clayton, Australia.
[4]Neuromedicines Discovery Centre, Monash University; Parkville, Australia.
[5]Division of Medicinal Chemistry, Leiden Academic Centre for Drug Research, Leiden University; Leiden, The Netherlands.
[6]Oncode Institute; Leiden, The Netherlands
[7]The Walter and Eliza Hall Institute of Medical Research; Parkville, Australia
[8]Department of Biochemistry and Pharmacology, The University of Melbourne; Melbourne, Australia
[†]These authors contributed equally to this work.

[*]Corresponding authors.
Contact at lauren.may@monash.edu, glukhova.a@wehi.edu.au, david.thal@monash.edu

The PDF file includes:

Figs. S1 to S10
Tables S1 to S3

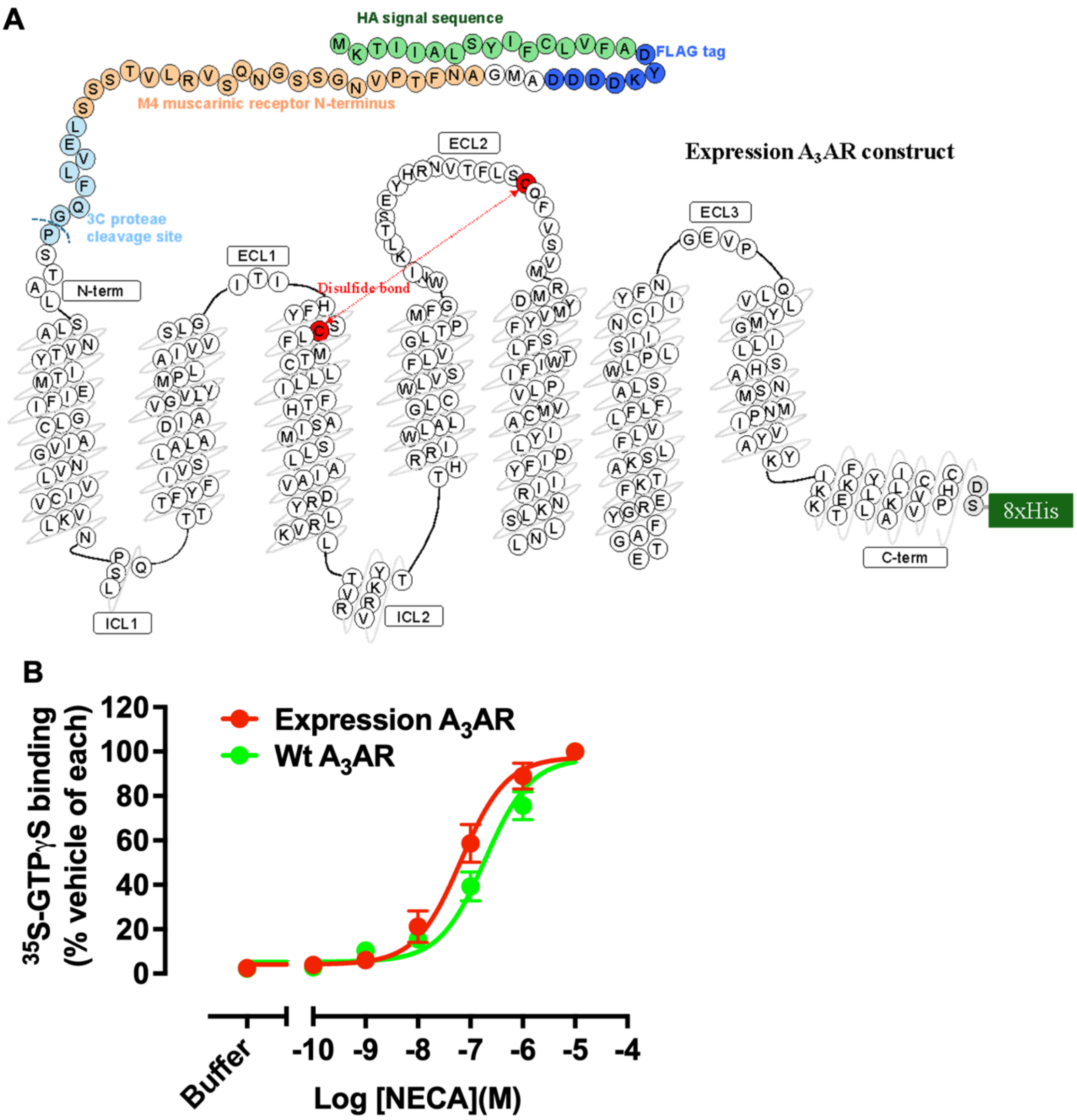

Fig. S1. Schematic and pharmacology of the A_3AR expression construct used in this study. **(A)** Snake plot of the A_3AR expression construct highlighting key features, including an N-terminal FLAG epitope followed by the M_4 mAChR N-terminus, a 3C protease cleavage site and a C-terminal 8X-His tag. **(B)** Comparison of [^{35}S]GTPγS binding between the expression A_3AR and WT A_3AR constructs. Data represent the mean ± SEM from n=3 experiments performed in quadruplicate. The potency values (pEC50) were 6.7 ± 0.1 (n=3) for WT and 7.2 ± 0.1 (n=3) for the expression A_3AR construct. The difference was statistically significant (P value = 0.03) using an unpaired t-test; however, receptor expression was not considered in these experiments, which could affect the potency values.

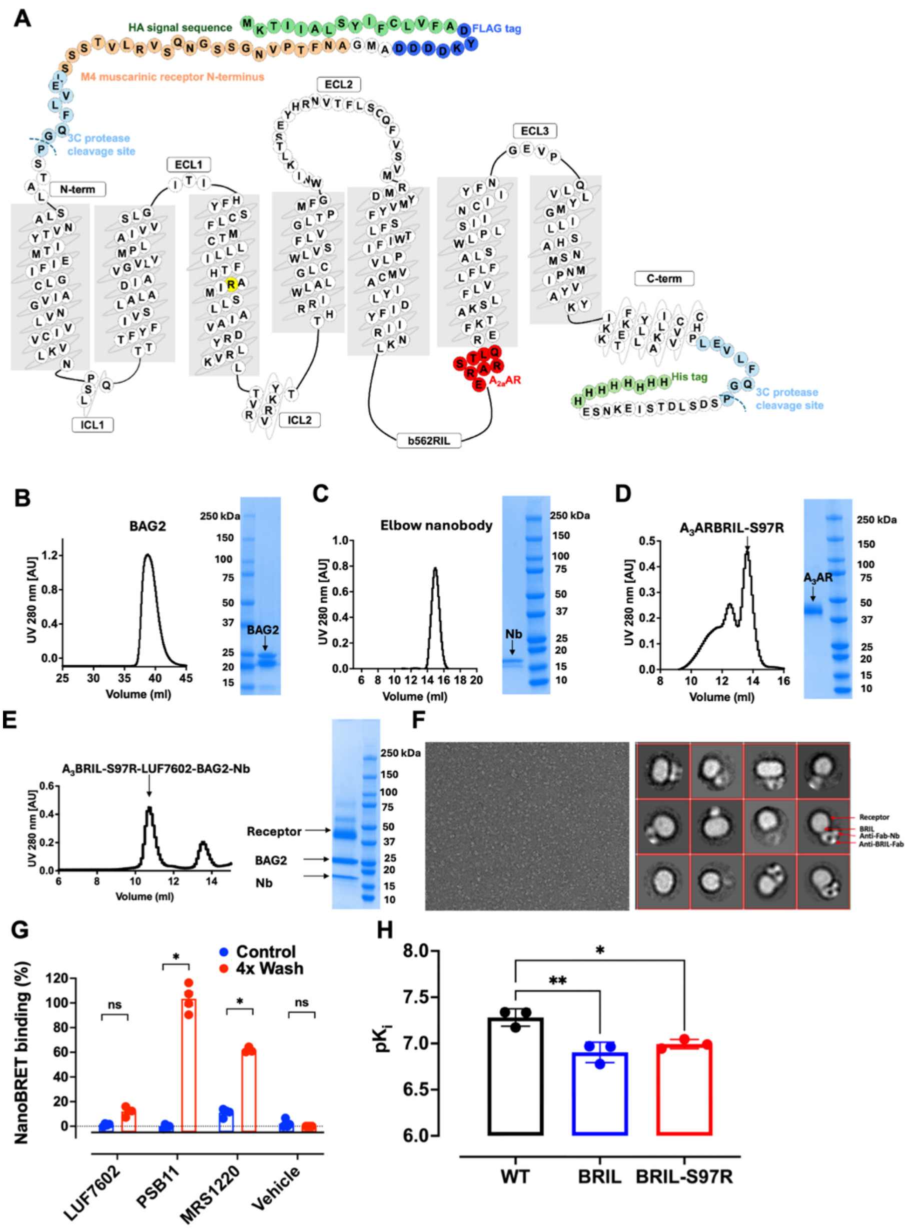

Fig. S2. Purification of the A_3AR-BRIL-S97R-LUF7602-BAG2-Nb complex.
(A) Schematic representation of A_3AR construct used to determine the inactive cryo-EM structure, which includes replacing ICL3 with BRIL and 8 residues of TM6 with the equivalent A_{2A}AR residues. **(B-E)** Size-exclusion chromatography profiles and corresponding SDS-PAGE gels for the purification of (B) the BAG2 chaperone, (C) the elbow nanobody (Nb), (D) A_3AR-BRIL-S97R, and (E) the A_3BRIL-S97R-LUF7602-BAG2-Nb complex. **(F)** Negative-stain electron microscopy images of the purified receptor complex showing a raw micrograph on the left and 2D class averages on the right. **(G)** Inhibition of XAC-630 in a NanoBRET binding assay comparing control (blue) and 4x wash (red) conditions for different antagonists (LUF7602, PSB11, MRS1220, and Vehicle). LUF7602 retained inhibition versus XAC-630 after a 4x washout, indicating irreversible binding. Data represent the mean ± SEM from n=3 experiments performed in triplicate. Statistics were performed using paired t-tests: ns = no significant difference; * = $P < 0.05$. **(H)** Bar graph showing pK_i values for WT, BRIL-S97R, and BRIL constructs in a competition NanoBRET binding assay with LUF7602. Significant differences were determined by one-way ANOVA (Prism 10.3.1) with a Dunnett's multiple comparison test; * = $P < 0.05$, ** = $P < 0.01$.

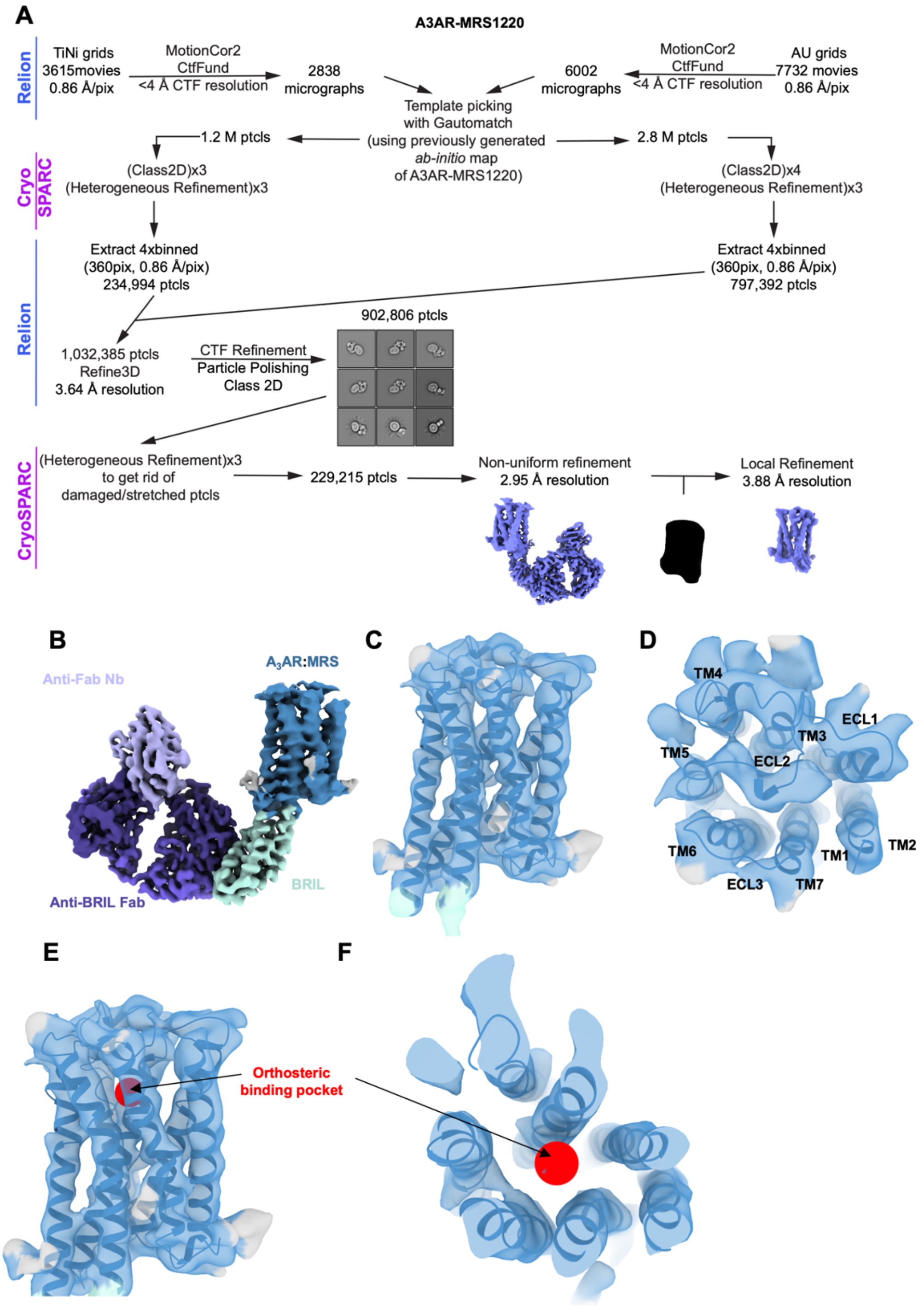

Fig. S3. Cryo-EM processing and EM map of the A_3AR-BRIL-S97R-BAG2-Nb complex purified with MRS1220.

(A) Cryo-EM data processing workflow for MRS1220-A_3AR. **(B)** 3D reconstruction of the A_3AR-BRIL-S97R-BAG2-Nb, showing the receptor (dark blue), BRIL fusion protein (cyan), anti-Fab nanobody (light purple), and anti-BRIL Fab (dark purple). **(C)** Cryo-EM density map of the A_3AR, showing the overall structure of the receptor and quality of the map. **(D)** Top view of the A_3AR EM map, highlighting the transmembrane helices (TM1-TM7) and extracellular loops (ECL1-3). ECL2 and the C-terminal portion of ECL1 could not be accurately modelled due to the lower resolution surrounding this area. **(E)** Side view and **(F)** top view of the A_3AR EM map with the adenosine binding site indicated by a red sphere. There was no EM density in the orthosteric binding pocket, which could be due to either MRS1220 not being bound or insufficient resolution to resolve the binding site.

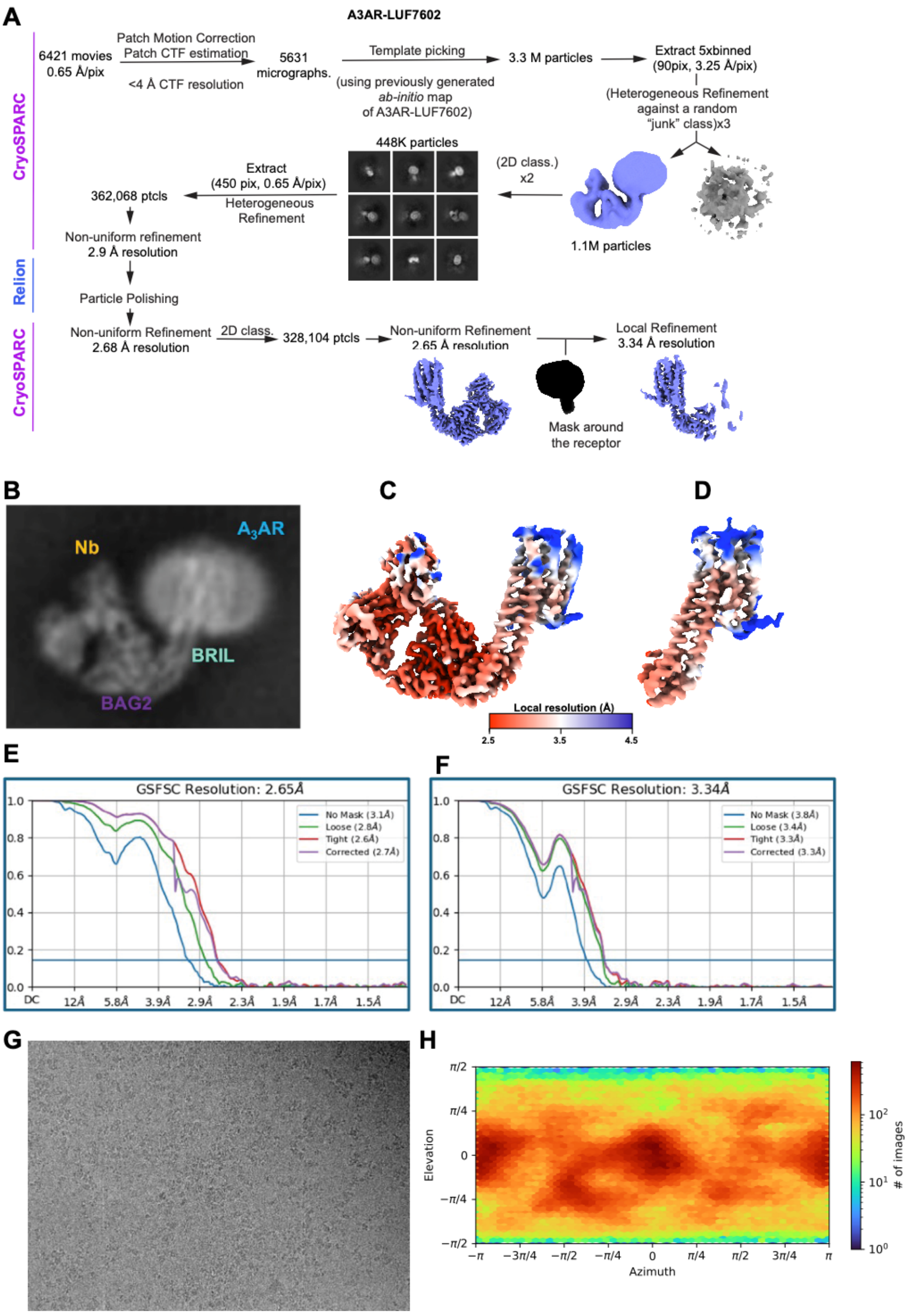

Fig. S4. Cryo-EM processing and EM maps of the LUF7602-bound to the A$_3$AR-BRIL-S97R-BAG2-Nb complex.

(A) Cryo-EM data processing workflow for LUF7602-A$_3$AR. **(B)** A 2D-class average of the complex in a detergent micelle viewed from the side. **(C-D)** Local resolution of the (C) consensus map and (D) the local-refined receptor map. **(E,F)** Gold-standard Fourier shell correlation (GSFSC) plot for (E) the consensus map and (F) the local-refined receptor map. **(G)** Representative micrograph. **(H)** A representation of the angular distribution of particles used in the final reconstruction.

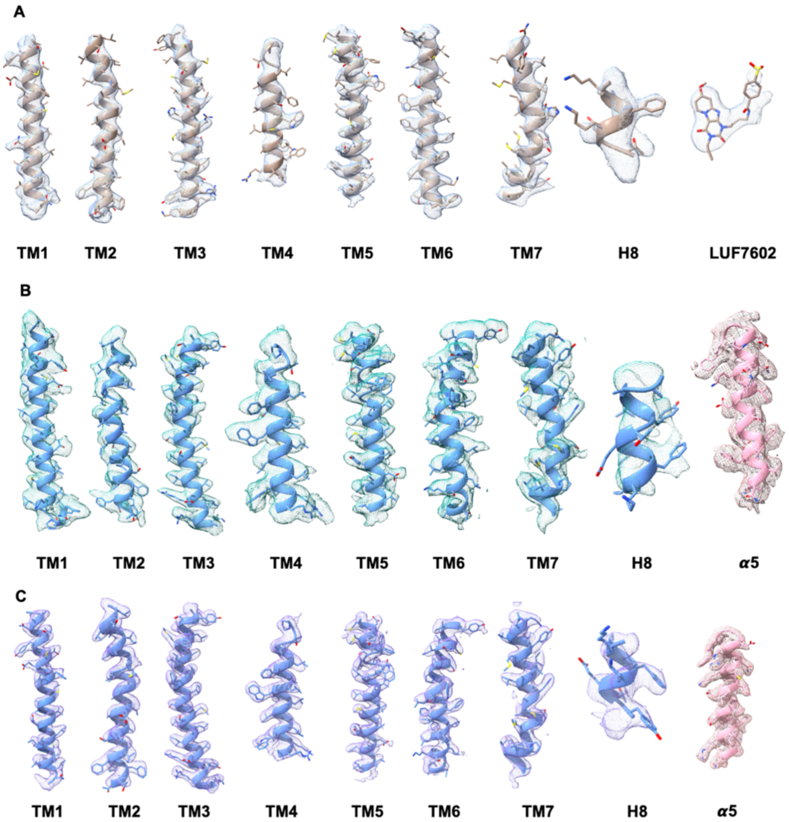

Fig. S5. Cryo-EM Density Maps

(A-C) Cryo-EM density maps of the seven transmembrane domains (TM1-TM7), helix 8 (H8) for the (A) LUF7602-bound (contour = 0.25), (B) adenosine-bound (contour = 0.3), and (C) Piclidenoson-bound (contour = 0.35) A_3AR complex structures. Panels (B,C) include the $\alpha5$ helix of the G protein.

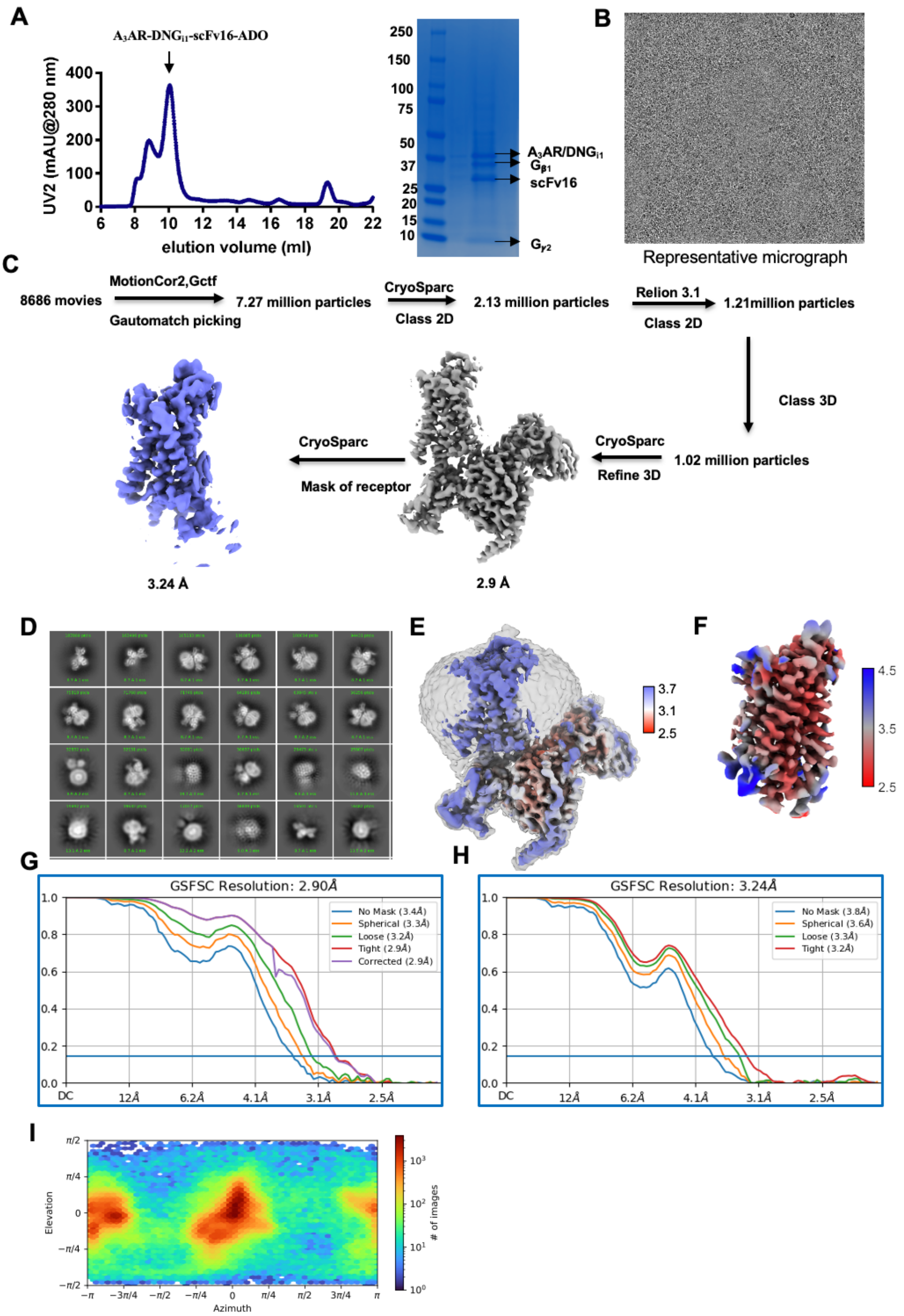

Fig. S6. Purification and cryo-EM processing of the adenosine-bound A_3AR complex. **(A)** Size-exclusion chromatography profile of purified A_3AR-DNG_{i1}-scFv16-ADO complex and SDS-PAGE gel showing the purified complex components. **(B)** Representative micrograph. **(C)** Cryo-EM data processing workflow for adenosine-A_3AR. **(D)** Reference-free 2D class averages of the complex particles. **(E)** Local resolution of the consensus map ranging from 2.5 Å (red) to 3.7 Å (blue). **(F)** Local resolution of the local-refined receptor map ranging from 2.5 Å (red) to 4.5 Å (blue). **(G,H)** Gold-standard Fourier shell correlation (GSFSC) plot for (F) the final consensus map and (G) the local-refined receptor map. **(I)** A representation of the angular distribution of particles used in the final reconstruction.

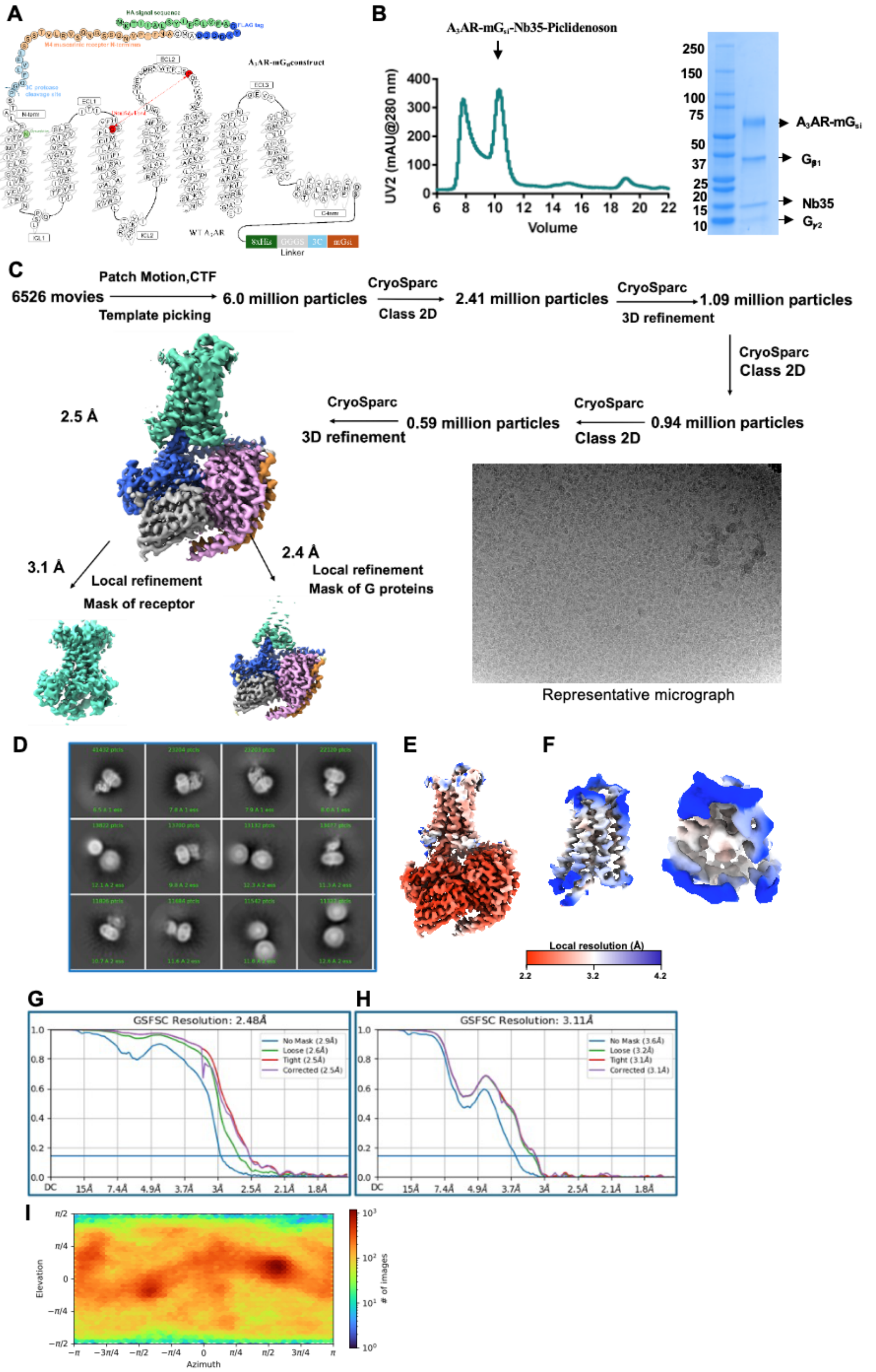

Fig. S7. Purification and cryo-EM processing of the Piclidenoson-bound A3AR complex. **(A)** Schematic representation of the A3AR-mG$_{si}$ construct. **(B)** Size-exclusion chromatography profile of purified A3AR-mG$_{si}$-Nb35-Piclidenoson complex and SDS-PAGE gel showing the purified complex components. **(C)** Cryo-EM data processing workflow for Piclidenoson-A3AR. **(D)** Reference-free 2D class averages of the complex particles. **(E)** Local resolution of the consensus map ranges from 2.2 Å (red) to 4.2 Å (blue). **(F)** Two views of the local resolution of the local-refined receptor map ranging from 2.5 Å to 3.5 Å. **(G,H)** Gold-standard Fourier shell correlation (GSFSC) plot for (G) the final consensus map and (H) the local-refined receptor map. **(I)** A representation of the angular distribution of particles used in the final reconstruction.

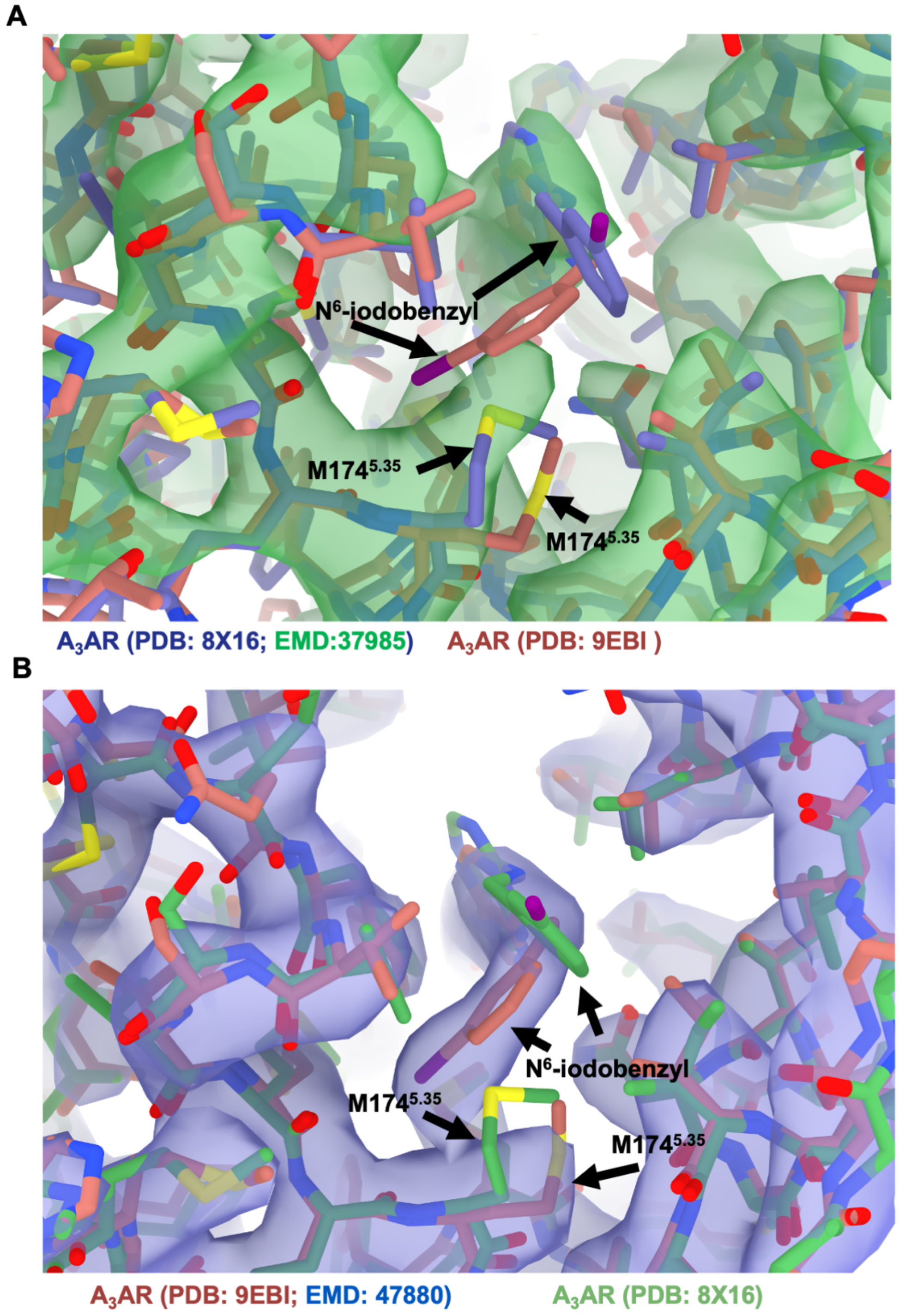

Fig. S8. Comparison of Piclidenoson-bound A_3AR structures

(A-B). Structural comparison of two A_3AR structures bound to Piclidenoson. The model of PDB: 9EBI is coloured peach in both panels. (A) The model of PDB: 8X16 is coloured blue, and the cryo-EM map is coloured green (EMD:37985, contour = 0.38). (B) The model of PDB: 8X16 is coloured green, and the cryo-EM map for PDB: 9EBI (EMD:47880) is coloured blue. The N^6-iodobenzyl group of Piclidenoson and key residue $M174^{5.35}$ is highlighted by arrows. There is a slight conformational difference in the position of residue $M174^{5.35}$ between the two structures, as shown by the cryo-EM maps. In the 8X16 structure, the position of $M174^{5.35}$ occludes the N^6-iodobenzyl group from a cryptic pocket created by ECL2 and TM5.

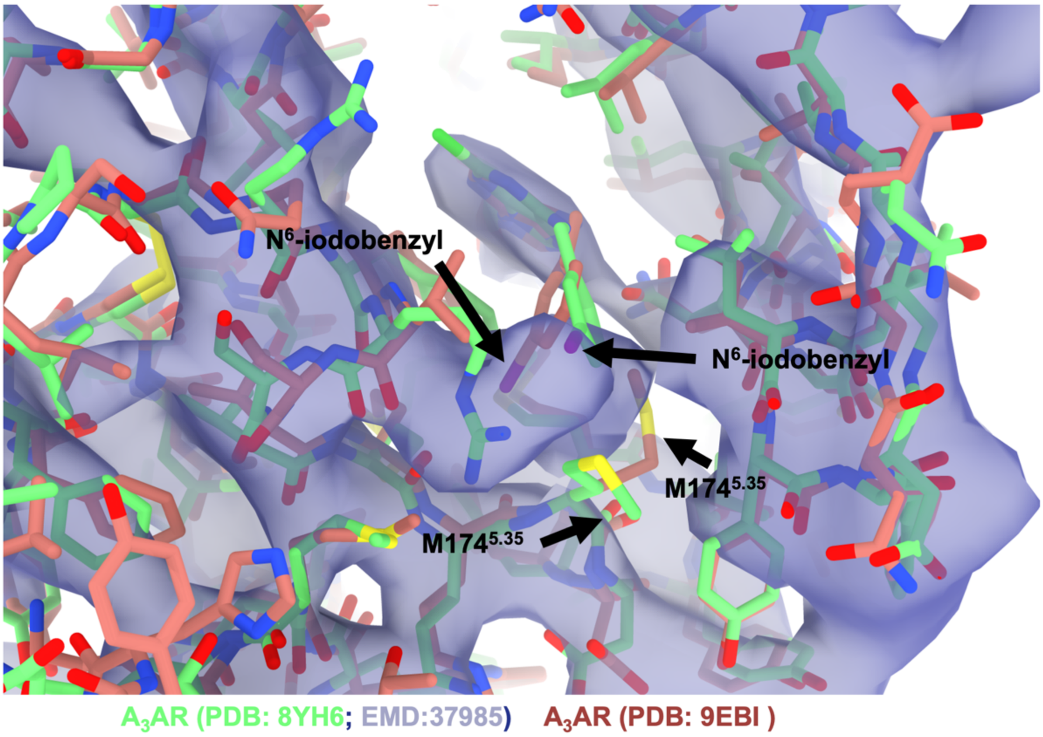

Fig. S9. Comparison of a Namodenoson-bound A_3AR structure

Similar to Fig. S8, a structural comparison of the Namodenoson-bound sheep A_3AR structure (PDB:8YH6) coloured green with cryo-EM density shown in blue (EMD: 37985, contour = 10) and our Piclidenoson-bound structure (PDB: 9EBI) coloured peach. The N^6-iodobenzyl groups and key residue M174$^{5.35}$ are highlighted by arrows. There is a conformational difference in the position of the N^6-iodobenzyl groups and residue M174$^{5.35}$ between the two structures.

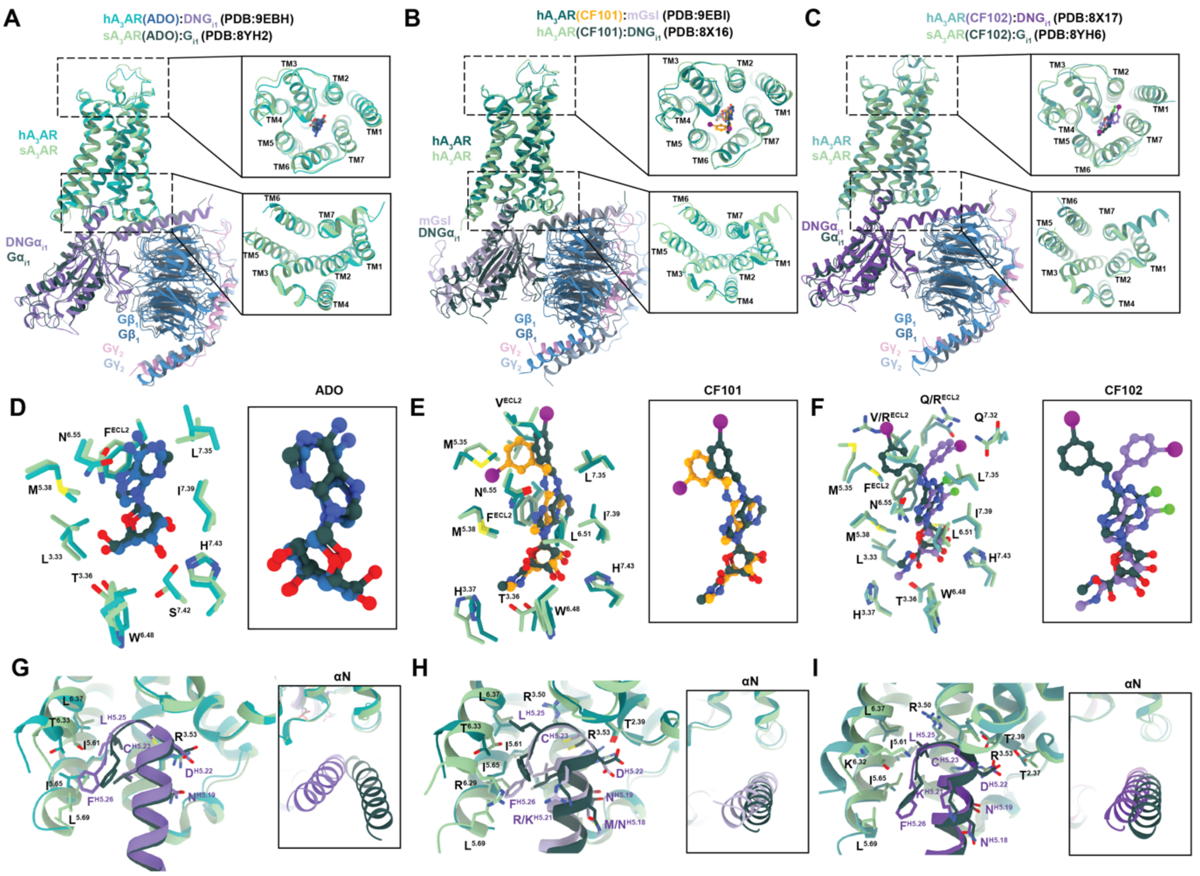

Fig. S10. Comparison of A_3AR structures from multiple studies

(A-C) Overview comparison of (A) the adenosine-bound sheep A_3AR and human A_3AR structures, (B) Piclidenoson-bound human A_3AR structures, and (C) Namodenoson-bound human A_3AR and sheep A_3AR structures. Insets include close-up views from the extracellular and intracellular sides.

(D-F) Related to A-C, a comparison of the orthosteric binding sites with insets comparing only the ligands.

(G-I) Related to A-C, a comparison of the G protein binding site with insets showing the αN helix of Gα.

Table S1. Cryo-EM data collection, refinement, and validation statistics

	A_3AR-DNGi1-scFv16-adenosine	A_3AR-mGsi-Nb35-Piclidenoson	A_3ARBRIL-BAG2-Nb-LUF7602
**Data Collection**			
PDB code	9EBH	9EBI	9EHS
EMD code (Consensus)<br>EMD code (Receptor Focus)<br>EMD code (Composite)	47879<br>47994<br>N/A	47880<br>47998<br>N/A	48063<br>48064<br>48065
Micrographs	8686	6526	6421
Electron Dose (e^-/A^2)	60	60	60
Voltage (kV)	200	300	300
Pixel size (Å)	0.99 and 1.03	0.82	0.65
Movie frames	50	60	60
Defocus range (µm)	0.5 – 1.5	0.5 – 1.5	0.5 – 1.5
**Refinement**			
Symmetry imposed	C1	C1	C1
Particles (final map)	325k	590k	328K
Resolution @0.143 FSC (Å)*<br>Complex<br>Receptor local refinement	 3.05<br>3.44	 2.48	 2.65<br>3.34
$CC_{map–model}$ (volume)	0.71	0.85	0.78
**Model Quality**			
R.M.S. deviations			
Bond length (Å)	0.003	0.005	0.004
Bond angles (°)	0.716	0.795	0.775
Ramachandran			
Favoured (%)	98.89	99.0	99.02
Outliers (%)	0	0	0
Rotamer outliers (%)	0	0.35	0
C-beta deviations (%)	0	0	0
Clashscore	4.02	4.90	2.86
MolProbity score	1.19	1.26	1.08

Table S2. Potency and E_{max} values from the $G\alpha_{i1}$ activation assay (TruPath)

**A_3 Adenosine Receptor**	**Adenosine** pEC_{50} (n)	**Piclidenoson** pEC_{50} (n)	**NECA** pEC_{50} (n)
**Y15A$^{1.35}$**	4.16 ± 0.36 (3)	6.50 ± 0.42 (3)	5.02 ± 0.02 (3)
**S73A$^{2.65}$**	5.90 ± 0.11 (3)	7.38 ± 0.03 (3)	6.73 ± 0.14 (3)
**T94A$^{3.36}$**	5.01 ± 0.13 (3)	6.76 ± 0.16 (3)	5.47 ± 0.10 (3)
**H95A$^{3.37}$**	3.57 ± 0.06 (3)	N.R. (4)	5.14 ± 0.07 (3)
**H95F$^{3.37}$**	N.R. (3)	N.R. (3)	N.R. (3)
**V169E$^{45.53}$**	6.64 ± 0.17 (3)	7.94 ± 0.01 (3)	7.40 ± 0.05 (3)
**M174A$^{5.35}$**	5.97 ± 0.12 (3)	8.16 ± 0.06 (3)	6.85 ± 0.06 (3)
**N250$^{6.55}$**	N.R. (3)	N.R. (3)	N.R. (3)
**Y265A$^{7.36}$**	6.01 ± 0.12 (3)	7.44 ± 0.07 (3)	7.05 ± 0.09(3)
**S271A$^{7.42}$**	3.19 ± 0.10 (3)	N.R. (4)	4.34 ± 0.15 (3)
**H272$^{7.43}$**	N.R. (3)	N.R. (3)	N.R. (3)
**A_3 Adenosine Receptor**	**Adenosine** ΔΔBRET Emax	**Piclidenoson** ΔΔBRET Emax	**NECA** ΔΔBRET Emax
**WT**	0.89 ± 0.08	0.78 ± 0.12	0.86 ± 0.10
**Y15A$^{1.35}$**	0.19 ± 0.02	0.21 ± 0.01	0.52 ± 0.06
**S73A$^{2.65}$**	1.49 ± 0.04	1.35 ± 0.04	1.51 ± 0.04
**T94A$^{3.36}$**	0.49 ± 0.04	0.21 ± 0.03	0.47 ± 0.01
**H95A$^{3.37}$**	0.63 ± 0.12	N.R	1.29 ± 0.04
**H95F$^{3.37}$**	N.R	N.R	N.R
**V169E$^{45.53}$**	0.67 ± 0.02	0.63 ± 0.06	0.72 ± 0.05
**M174A$^{5.35}$**	1.35 ± 0.01	1.29 ± 0.04	1.33 ± 0.04
**N250$^{6.55}$**	N.R	N.R	N.R
**Y265A$^{7.36}$**	1.25 ± 0.04	1.23 ± 0.01	1.41 ± 0.02
**S271A$^{7.42}$**	0.70 ± 0.15	N.R	0.35 ± 0.06
**H272$^{7.43}$**	N.R	N.R	N.R

Mean ± SEM pEC_{50} and maximum response (Emax) values for adenosine, NECA, and Piclidenoson from HEK293A cells transiently expressing WT A_3AR and single residue mutants. N.R. denotes mutants in which a measurable response could not be detected. Values are calculated from three independent experiments conducted in duplicate.

Table S3. $G\alpha_{i1}$ activation assay (TruPath)

A_3 Adenosine Receptor	Adenosine log τ (n)	Piclidenoson log τ (n)	NECA log τ (n)
**WT**	1.6 ± 0.2 (3)	0.7 ± 0.1 (3)	1.1 ± 0.2 (3)
**Y15A$^{1.35}$**	0.6 ± 0.3 (3)	0.8 ± 0.3 (3)	N.D. (3)
**S73A$^{2.65}$**	1.4 ± 0.07 (3)	0.8 ± 0.07 (3)	1.4 ± 0.07 (3)
**T94A$^{3.36}$**	0.8 ± 0.09 (3)	0.01 ± 0.07 (3)	1.2 ± 0.1 (3)
**H95A$^{3.37}$**	0.3 ± 0.3 (3)	N.D. (3)	3.9 ± 0.02 (3)
**H95F$^{3.37}$**	N.R. (3)	N.R. (3)	N.R. (3)
**V169E$^{45.53}$**	1.6 ± 0.1 (3)	0.4 ± 0.09 (3)	1.6 ± 0.1 (3)
**M174A$^{5.35}$**	-0.1 ± 0.2 (3)	-0.1 ± 0.2 (3)	-0.1 ± 0.2 (3)
**N250A$^{6.55}$**	N.R. (3)	N.R. (3)	N.R. (3)
**Y265A$^{7.36}$**	1.4 ± 0.07 (3)	0.8 ± 0.06 (3)	2.1 ± 0.07 (3)
**S271A$^{7.42}$**	N.D. (3)	N.R. (3)	N.R. (3)
**H272A$^{7.43}$**	N.R. (3)	N.R. (3)	N.R. (3)

Data are the mean ± SEM values from three experiments performed in duplicate with the log τ determined from a global fit of the data using the operational model of agonism using K_A values from **Table 2** and then corrected for receptor expression relative to WT using B_{max} values from **Table 1**. N.D. = efficacy not determined due to lack of binding affinity data from **Table 2**. N.R. = no measurable response.
