# Supplementary material for: Molecular basis of ligand binding and receptor activation at the human A3 adenosine receptor
Source: Nat Commun. 2025 Aug 18;16:7674. doi: 10.1038/s41467-025-62872-x (PMC12361428; doi:10.1038/s41467-025-62872-x)
Supplement: Supplementary file 2 — Reporting Summary [file 41467_2025_62872_MOESM2_ESM.pdf]

nature portfolio

Corresponding author(s): Thal, DM

Last updated by author(s): Jul 17, 2025

Reporting Summary

Nature Portfolio wishes to improve the reproducibility of the work that we publish. This form provides structure for consistency and transparency in reporting. For further information on Nature Portfolio policies, see our Editorial Policies and the Editorial Policy Checklist.

Statistics

For all statistical analyses, confirm that the following items are present in the figure legend, table legend, main text, or Methods section.

n/a	Confirmed	
☐	☒	The exact sample size (*n*) for each experimental group/condition, given as a discrete number and unit of measurement
☐	☒	A statement on whether measurements were taken from distinct samples or whether the same sample was measured repeatedly
☐	☒	The statistical test(s) used AND whether they are one- or two-sided<br>*Only common tests should be described solely by name; describe more complex techniques in the Methods section.*
☒	☐	A description of all covariates tested
☒	☐	A description of any assumptions or corrections, such as tests of normality and adjustment for multiple comparisons
☐	☒	A full description of the statistical parameters including central tendency (e.g. means) or other basic estimates (e.g. regression coefficient) AND variation (e.g. standard deviation) or associated estimates of uncertainty (e.g. confidence intervals)
☐	☒	For null hypothesis testing, the test statistic (e.g. *F*, *t*, *r*) with confidence intervals, effect sizes, degrees of freedom and *P* value noted<br>*Give P values as exact values whenever suitable.*
☒	☐	For Bayesian analysis, information on the choice of priors and Markov chain Monte Carlo settings
☒	☐	For hierarchical and complex designs, identification of the appropriate level for tests and full reporting of outcomes
☒	☐	Estimates of effect sizes (e.g. Cohen's *d*, Pearson's *r*), indicating how they were calculated

Our web collection on statistics for biologists contains articles on many of the points above.

Software and code

Policy information about availability of computer code

Data collection	Data collection for cryo-EM data was done with SerialEM.
Data analysis	GraphPad Prism 10, Relion 3.1, CryoSPARC 2.15, 3.1, Coot-0.9, PyMol-2.5, Chimera-1.18, ChimeraX-1.9, Phenix-1.21, and MolProbity v4.

For manuscripts utilizing custom algorithms or software that are central to the research but not yet described in published literature, software must be made available to editors and reviewers. We strongly encourage code deposition in a community repository (e.g. GitHub). See the Nature Portfolio guidelines for submitting code & software for further information.

Data

Policy information about availability of data

All manuscripts must include a data availability statement. This statement should provide the following information, where applicable:

- Accession codes, unique identifiers, or web links for publicly available datasets
- A description of any restrictions on data availability
- For clinical datasets or third party data, please ensure that the statement adheres to our policy

Atomic coordinates for the A3AR-BRIL-BAG2-Nb-LUF7602, A3AR-DNGi1-scFv16-adenosine, and A3AR-mGsi-Nb35-Piclidenoson complex structures were deposited in the Protein Data Bank (PDB) with the accession codes 9EHS, 9EBH, and 9EBI, respectively. Cryo-EM maps were deposited in the Electron Microscopy Data Bank under the accession codes EMD-47879 (consensus map) and EMD-47994 (receptor focus map) for the A3AR-DNGi1-scFv16-adenosine complex, EMD-47880

(consensus map) and EMD-47998 (receptor focus map) for the A3AR-mGsi-Nb35-Piclidenoson complex, and EMD-48063 (consensus map) and EMD-48064 (receptor focus map), EMD-48065 (composite map) for the A3AR-BRIL-BAG2-Nb-LUF7602 complex. Source data are provided with this paper.

Research involving human participants, their data, or biological material

Policy information about studies with human participants or human data. See also policy information about sex, gender (identity/presentation), and sexual orientation and race, ethnicity and racism.

Reporting on sex and gender	N/A
Reporting on race, ethnicity, or other socially relevant groupings	N/A
Population characteristics	N/A
Recruitment	N/A
Ethics oversight	N/A

Note that full information on the approval of the study protocol must also be provided in the manuscript.

Field-specific reporting

Please select the one below that is the best fit for your research. If you are not sure, read the appropriate sections before making your selection.

☒ Life sciences ☐ Behavioural & social sciences ☐ Ecological, evolutionary & environmental sciences

For a reference copy of the document with all sections, see nature.com/documents/nr-reporting-summary-flat.pdf

Life sciences study design

All studies must disclose on these points even when the disclosure is negative.

Sample size	Sample sizes were typically n=3 to n=5 to ensure sufficient data for identifying statistical significance between samples.
Data exclusions	For cyro-EM data analysis, micrographs with low estimated CTF resolution were excluded from processing.
Replication	No replication was performed for structural biology studies, as in standard in the field. For pharmacology assays, each individual experiment was performed in duplicate or triplicate.
Randomization	No randomization was performed for pharmacology experiments, though plate layouts were altered between experiments. No animals or human research participants were involved in this study.
Blinding	Blinding was not performed in this study. No animals or human research participants were involved in this study.

Reporting for specific materials, systems and methods

We require information from authors about some types of materials, experimental systems and methods used in many studies. Here, indicate whether each material, system or method listed is relevant to your study. If you are not sure if a list item applies to your research, read the appropriate section before selecting a response.

Materials & experimental systems

n/a	Involved in the study	
☐	☒	Antibodies
☐	☒	Eukaryotic cell lines
☒	☐	Palaeontology and archaeology
☒	☐	Animals and other organisms
☒	☐	Clinical data
☒	☐	Dual use research of concern
☒	☐	Plants

Methods

n/a	Involved in the study	
☒	☐	ChIP-seq
☒	☐	Flow cytometry
☒	☐	MRI-based neuroimaging

Antibodies

Antibodies used	Nanobody 35, scFv16, anti-BRIL Fab, and the anti-Fab nanobody were expressed and purified in house. Mouse poly-His antibody was purchased from QIAGEN.
Validation	Nanobody 35, scFv16, anti-BRIL Fab, anti-Fab nanobody were validated by experimental structure. Mouse poly-His antibody was used on purified protein samples that contained a His tag.

Eukaryotic cell lines

Policy information about cell lines and Sex and Gender in Research

Cell line source(s)	Sf9 and TNI cells were purchased from Expression Systems. CHO cell lines were purchased from Thermo Fisher Scientific.
Authentication	Cells were grown from original stocks purchased from suppliers. They were not authenticated in house.
Mycoplasma contamination	CHO cells were regularly tested to ensure they were free from mycoplasma contamination. Sf9 and TNI cells were not tested.
Commonly misidentified lines (See ICLAC register)	No commonly misidentified cell lines were used.

Plants

Seed stocks	N/A
Novel plant genotypes	N/A
Authentication	N/A
